# Exploring the Implications of Aged Care Reform on Allied Health Workforce and Capacity to Deliver Palliative and End-of-Life Care

**DOI:** 10.3390/healthcare13243207

**Published:** 2025-12-08

**Authors:** Olivia Farrer, Jennifer Tieman

**Affiliations:** Research Centre for Palliative Care, Death and Dying (RePaDD), College of Nursing and Health Sciences, Flinders University, Bedford Park, SA 5042, Australia; jennifer.tieman@flinders.edu.au

**Keywords:** allied health, residential aged care, funding, older adults

## Abstract

**Background**: The aged care sector in Australia is increasingly focused on a personalised care approach which prioritises dignity and respect for older adults. With a growing ageing population, we are also seeing more complexity in health needs and palliative care. Allied health professionals are skilled in the delivery of care that fosters independence and quality of life as health deteriorates. However, in the recent aged care reform, there has been concern that allied health are not clearly recognised for their role in aged and palliative care. **Methods**: Qualitative study using semi-structured interviews with aged care service providers and allied health peak body representatives and clinicians to understand the perceived impact of aged care reforms in Australia on allied health workforce. Guided by a constructivist epistemology, reflexive thematic analysis was used to understand key themes. **Results**: Eight interviews were conducted, yielding one key theme and three sub themes. These were person-centred palliative care, advocates, location of care matters, and funding model, with the key theme of the funding model intersecting with all other themes. **Conclusions**: The issue of recognising allied health best practice in aged and palliative care is multifactorial. At the heart of the problem appears to be how well recognised allied health disciplines are for the value of their contribution, and constraints imposed by funding models and sector priorities on the sufficient input of the full scope of practice. Without a sufficient and skilled workforce supported to enact their full scope, the ability to deliver a personalised care approach and outcomes for our ageing population will suffer.

## 1. Introduction

Globally, we are confronted with a growth in the ageing population, as people are living longer [1]. It is estimated that by 2026, more than 22% of Australians will be aged over 65 years. By 2063, it is predicted that this will rise again and that for every five working Australians there will be almost two older people [2], and this is a story seen worldwide, particularly in developed countries. Living for longer offers opportunities for the older person and their families, as they can pursue interests and continue to contribute to the family, the economy, and their society. However, the rising number of older adults is also likely to increase the country’s health spending, and resourcing for aged care services such as maintaining agency and independence will require proactive health management [1]. In Australia, the anticipated demand has implications for a sector where there is already a shortage of care workers [3] and a chronically underfunded aged care system [4].

The aged care sector in Australia is increasingly focused on a humanistic and personalised care approach which prioritises dignity, choice, and respect for older adults within the system [4]. Delivering this type of care is complex and will present challenges in creating opportunities and capability for the aged care workforce to engage with older adults sufficiently to deliver personalised care. Almost one in three older Australians are living with three or more chronic diseases (multimorbidity) [5]. This increases the complexity of health needs and has a significant impact on frailty and functional capacity, ultimately creating an unpredictable health trajectory [6]. For all older adults, death can be seen as a foreseeable event. While not all older adults in aged care may require specialist palliative care, older people living with one or more progressive diseases would likely benefit from a palliative approach to improve quality of life and address end-of-life planning and individualised care needs [6,7].

Palliative care has traditionally been thought of as the care of people with a life-limiting illness. Recent reframing sees the approach re-defined as an intent to relieve “serious health related suffering, be it physical, psychological, social, or spiritual,” with the cause of suffering being extended to a range of chronic health conditions or “extreme frailty of old age” and available at all levels of care [7]. The World Health Organization (2020) goes on to say that evidence supports that a palliative care approach offers a support system to help people live as actively as possible until death [7]. This conceptualization is consistent with the individualised care approach described in the newly strengthened Aged Care Quality Clinical Standards (from November, 2025) [8]. It is likely that most older adults in receipt of aged care services could benefit from a palliative approach. This requires a sufficient and capable health and ageing workforce and a system that enables the delivery of such care [9].

The coordination and delivery of palliative and individualised care can be undertaken by a wide range of health professionals. Allied health describes a group of health professionals [10] who have expertise in working within an interprofessional team and offer diverse skills related to assessment, diagnosis, treatment, and counselling [11]. They actively respond and address specific palliative care needs and can contribute to the coordination role of palliative care. Currently, allied health makes up over a quarter of registered health professionals in Australia; however, less than 5% are thought to be working in residential aged care [11]. The peak body for allied health, Allied Health Professions Australia (AHPA), are concerned that this workforce will be further negatively impacted by the recent aged care reforms [12]. In fact, a survey by AHPA (2023) found that in the months since the reform began, one in eight allied health professionals had lost their aged care employment, and a further 30% were planning to leave the sector [13]. The aged care sector is complex and the interplay between funding models and allied health service provision in aged care is multifaceted.

End of Life Directions for Aged Care (ELDAC) is a project funded by the Australian Government Department of Health, Disability and Ageing to support quality care for older Australians at the end of life. In particular, the ELDAC Allied Health Toolkit was developed to support allied health clinicians in this sector [14]. The stakeholder interviews discussed in this study were undertaken to understand how allied health professionals interact with aged care providers and service users, and to further understand the impact funding changes may have and how the allied health toolkit might support practice within the current aged care environment. This report provides a synthesis of interviews conducted with representatives from peak bodies, aged care providers, health professional groups and perspectives from allied health clinician experiences of working with older adults requiring palliative and end-of-life (EOL) care, in aged care.

## 2. Materials and Methods

This study used a qualitative methodology to explore the lived experience and professional opinion of a purposeful sample of key stakeholders with experience of how allied health practitioners interact with the Australian aged care system broadly and then specifically to palliative care. The study acknowledges the Consolidated Criteria for Reporting Qualitative Research (COREQ) in reporting on this study. The project team was small, with the study being a discrete activity within a larger project [14]. The two investigators had varied research experience, comprising a research fellow with an allied health clinical background (OF), and a professor with significant experience in palliative and aged care, implementation and knowledge translation (JT). The study was approved by Flinders University Human Research Ethics committee, project number 5680.

### 2.1. Recruitment

Participant health professionals, peak body representatives, and aged care service providers were a convenience sample that was purposively recruited through an open invitation email to participate in the study. The email included the participant information and consent form for further information, and the project lead (OF) was also available to discuss any project details in more detail. A total of twelve invitations were sent to allied health peak bodies, aged care providers (residential and home-based care) and researchers that had previously engaged with members of the project team in working parties for other project activities, or who were actively involved in allied health advocacy work. Participants indicated interest in participating by replying to the email and returning a completed consent form. Participants were advised that interview responses would not be identifiable, although they may recognise their own responses.

### 2.2. Data Collection

**Figure 1:**
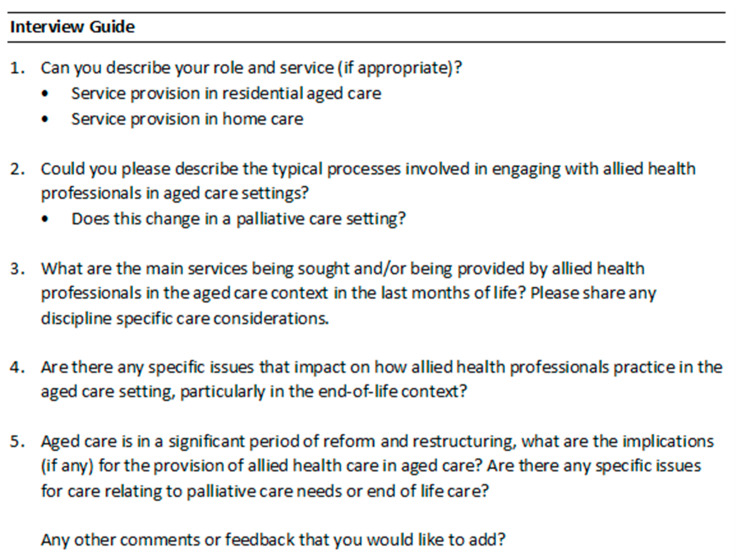
Interview guide used in semi-structured interviews.

Within the qualitative methodology, semi-structured interviews were conducted by one of the project investigators (OF) to collect open-ended data in the form of participant perspectives. Interviews were conducted using an interview guide (Figure 1) that had been developed by the project team and approved in the project ethics. One investigator (OF) conducted all interviews using MS Teams videoconferencing. The interviews were recorded and transcribed verbatim, and interviewees were invited to provide additional comments to their interview transcripts if wanted. Data were saved in a university managed research drive, which was password-protected and had limited access to project researchers only, and deleted from MS Teams.

### 2.3. Analysis

Analysis of the interviews was guided by the six steps of reflexive thematic analysis recommended by Braun and Clarke (2019) [15]. Reflexive thematic analysis is considered to be a reflection of the researcher’s interpretive analysis of the data conducted at the intersection of the following: (1) the dataset; (2) the theoretical assumptions of the analysis, and (3) the analytical skills/resources of the researcher. In this method, coding can be completed by one person and should be reflective engagement with the data. Subsequently, the second author (JT) ‘sense-checked’ theming to achieve a richer interpretation of the data, rather than attempting to achieve consensus.

This method allows for a more organic approach to analysis, and themes are produced by organising codes around a ‘central organizing concept’ [15]. The reflexive thematic analysis approach still incorporates six steps: familiarisation with the data, generating initial codes, generating themes, reviewing potential themes, defining and naming themes, and producing the report [15]. The interview questions for this study were addressed within a constructivist framework, which aimed to explore stakeholder perceptions and experiences of the aged care reform, directly aligning with the research question.

## 3. Results

Interviews were conducted with four allied health peak body organizations, two research organizations with an aged and palliative care focus, two aged care organizations, a primary health network representative, and incorporated clinician perspectives from podiatry, occupational therapy, speech pathology, and dietetics. A total of eight interviews were conducted (with five interviewees representing their peak or research body, as well as offering a professional opinion as a clinical allied health practitioner). Interviews lasted between 30 and 60 min, the timing tending towards the 60 min where a participant was representing a peak body, and clinician perspective (often in the form of lived experience exemplars) in their responses. All but one of the interviewees (representing an aged care provider) were female. The two aged care providers represented organizations with service reach across Australia in a collective n = 42 residential aged care sites (metro and rural) across South Australia, New South Wales, Victoria, Queensland, and Western Australia (Table 1).

On reviewing the data, the project team saw significant integration within codes and themes, comprising one key theme and three sub-themes. These were person-centred palliative care, advocates and location of care matters, and funding model, with the key theme of funding model intersecting with all other themes. Table 2 provides an overview of the themes and sub-themes, including an exemplar quote for each.

**Sub-Theme 1, Person-Centred Palliative Care:** “one of their first questions … was what’s important to you now?” (P4)

Person-centred palliative care should comprise a collaborative health care team that has time to identify personal goals and adjust across the living and dying trajectory of ageing, and should include opportunities for the individual to express their preferences and wishes for end-of-life care [9]. The participants interviewed in this study were, unsurprisingly, in consensus in describing palliative and end-of-life care as needing to be person-centred; however, they acknowledged that enacting this is complex. Providing person-centred care relies on the broader health team being invited to be part of care; sufficient resourcing (time and funding) for initial and ongoing care; and a confident workforce to deliver the care. The workforce also needs to be able to foster trusting and effective clinical relationships across the care trajectory, including at the end of life. A concern raised by several of the participants was that without specific allocated funding for allied health input in aged care for proactive reablement care, there is also a risk that services may not have teams that can support palliative and end-of-life care:

“… *the whole concept of advanced care planning is still sadly nowhere near as common as it should be in terms of having the right conversations around what matters most and maximizing quality of life…And we’re not talking about the last days or weeks and pain management, this is a much bigger conversation, and it starts generally when they walk through the door* … *or enroll with your service from home*…”.

(P8)

There was also a broad concern that not only might referrals be less forthcoming, but the proposed strengthened aged care standards [8] and associated funding may also limit the ability for allied health disciplines to enact their full scope or attract sufficient funded time for their services. Consequently, in the first instance, the individual will be impacted, with timely input not being received. More broadly, there are implications for the allied health workforce, who already see working in aged care as being less clinically challenging or rewarding and consequently are leaving the aged care workforce [13]. Several stakeholders also acknowledged the risk of losing permanent allied health roles and opting instead for ad hoc contracted allied health services. Such decisions could lead to a higher turnover of new graduates “*cutting their teeth in aged care because there’s so much work but not lasting very long*” (P3).

**Sub-Theme 2, Advocates**

When allied health input to aged and palliative care is initiated for an older person, the funding body that pays for at least part of it relies on the advocacy of others, the older person, or even the allied health professional themselves, to navigate the health and aged care systems. Aged care funding models are complex, and the proposed strengthened aged care standards [8] at the time of this study did not name specific allied health services that should be involved in the multidisciplinary health team. This creates risk for disparity in the way that allied health professionals could be engaged in palliative aged care, “*And I think you know the big takeaway there is that, you know, the funding mechanisms have recognized some professions if you like and not others… And when they’re not recognized, we’re not funded in a per se to deliver those*…” (P1).

Where an older adult cannot advocate for their own allied health services, the person responsible for initiating allied health referrals (or ‘gatekeeper’) should ideally have a sufficient understanding of what value allied health professions might contribute and facilitate in timely and sufficient referrals. However, at present, it is more usual that allied health is used in an ‘ad hoc’ way, “*… some of them will just have a regular schedule that seems to work well because it also prompts referrals like the nursing staff and know that*… *this for instance the podiatrist is coming in next week… So, they’ll identify anyone else that needs to be seen, and then there are other services that…will come in as required*” (P1).

There was broad concern amongst the allied health professional interviewees that this will create a ‘deficit approach’ to care, where allied health care is only initiated once overt symptoms and problems occur, rather than as a proactive measure, supporting reablement and maintaining function and independence for as long as possible, “…*you know, we would expect referrals to speech pathology if someone has swallowing is deteriorating or we need to upgrade their diet… and do see referrals for OT and Physio but that becomes more around manual handling and pressure care*…” (P1).

If the individual is receiving aged care services in their own home, they may have family, carers, and a GP and/or homecare coordinator to advocate on their behalf for adequate funding for allied health services, “*The individuals who have got families that care and are perhaps more involved in them*… *in aged care facilities and the community as well, who have got money and the means to get in a private therapist, certainly do better and get more of an input.*” (P3). However, timely and adequate allied health input will still require these referrers to understand how various allied health disciplines can support the older person, and this can be challenging when in aged and palliative care, the focus is less on improving clinical outcomes and more around ‘*adjusting to deterioration*’ and quality of life. “*You may stabilize, improve for short time, but everybody’s going to deteriorate no matter what you do*” (P4).

**Sub-Theme 3, Location of Care Matters**

Aged care providers only spoke to the residential setting, but all other stakeholders generally agreed that there would be differences in the volume and type of allied health input and palliative care between settings when comparing residential to home-based aged care. They proposed that this could be further exacerbated by state-based variation, and how acute care and aged care services relate to each other, “…*the aged care sector is doing it really tough and I think in terms of being a really key and very equal partner in terms of a care provider <they> often don’t have a voice or often don’t feel as connected as they could be in terms of discharge planning, getting the right information to really design person centered care*” (P8).

Overall, there appears to be disparity in the way individuals are managed, and how the systems relate to each other in terms of who funds what input and in what care context. An example of the movement between acute care to aged care services highlighted potential care discrepancies:

“…*in reality, if you live in residential aged care, your input and your likelihood of receiving rehabilitation is much reduced*… *So if you fall at home and you’re living at home and you go into hospital and have … your hip fracture fixed, you will receive rehabilitation and home care to get you back home… If you fracture your hip and you’re living in residential aged care, you’ll go and get that hip fixed in hospital and then potentially be discharged back to your residential care facility with no additional support*… *if you’re living in residential aged care, then why should the hospital fund that rehabilitation?*… *it’s sort of seen as double dipping if you’re trying to get rehabilitation when you’re already living in a high-level care service*”.

(P3)

Several stakeholders were more optimistic about the ability to deliver person-centred care in community-based aged care services; however, they noted that there is still a risk of inconsistency because of the sub-contractor model and high turnover of new graduates employed in this space:

“*Home care services are now being delivered quite well. So it’s certainly better than the residential aged care facilities, but again, you do have that potential for that variability of you know, not seeing the same person each week depending on who gets rostered on in that area and who’s available. So that inconsistency, I think it’s probably across the board*”.

(P3)

Further complicating the issue of access to allied health services, several stakeholders also noted that for older adults located in rural and remote areas, access would likely be further limited to the availability and capacity of local services, creating significant variability in the care experience, “… *a couple of our rural locations where we’ve been unsuccessful recruiting internally for an OT or physio… so we do engage in OT or physio* … *externally for those 2 sites*” (P1).

**Major Theme, Funding Model** “*And when they’re not recognized, we’re not funded in a per se to deliver those and it becomes* …*very difficult*…” (P1).

The consensus from all stakeholder interviews was that the level of engagement with allied health services and the delivery of care, whether it be reablement or palliative care, is primarily determined by how the service is funded; this theme intersected all others. The approach to aged care has changed to focus on quality of life and maintaining function, in a reablement and restorative approach. While allied health professions are typically familiar with the implementation of these approaches in residential aged care, the relationship to palliative care may not be recognised or prioritised.

One stakeholder noted that there is still a mindset in primary health care and aged care that considers palliative care and end-of-life care almost as last days. There was concern that palliative approaches are poorly understood and so not well implemented, despite the fact that, “…*palliative care is still very much part of living and it is the core business of aged care*” (P8). Moving forward, it will be important that these approaches are enabled at the service level and draw upon named funding streams supporting them,

“*And I think even the terminology so some of the terminology suggests that people living in aged care can’t get funding for rehabilitation. But reablement and restorative care they can, and it just comes down to the terminology in some of the government documentation*”.

(P3)

Similarly, the importance of consistency in how definitions are understood and enacted was raised again in how we discuss allied health. A clinician and research stakeholder proposed that using the general term ‘allied health’ when referring to many disciplines with unique offerings towards palliative care may be doing the professions a disservice in creating a blurred scope of practices: the risk being that our individual roles are poorly understood, the value of having a particular discipline deliver the role is not recognised, and thus not acknowledged separately or with reference to specific activities in funding bodies,

“*the phrase allied health, I think, is potentially a dangerous one, because I think it hides the different disciplines and we’re not the same” and, “*…*it has direct links for consumers as well, because if you say ‘well I need a walking frame’…who can help or teach me how to…get up, on and off safely … in subacute, in rehab everybody knew what the OT did*…*what the physio did, what the speech path did…you were used to capacity*”.

(P4)

Advocacy for funding to enact full scope of practice will somewhat rely on the various allied health disciplines to advocate for their role in palliative and end-of-life care in aged care. With reference to scope of practice, stakeholders interviewed in this study identified that the role of allied health in palliative care is more extensive than the discipline-specific services. They proposed that clients value having health team members that they can trust, and through these relationships, holistic care conversations often occur:

“…*the more vulnerable populations you know, building that trust, that sense of trust to, you know, let someone into your home. I think is a big deal, so having people you know, rolling through who you’ve got no idea who they are. Yeah. Is it really impacts care*”.

(P4)

So much of palliative care sits around clinical reasoning, client-centred care, confidence in EOL conversations, and communicating with the broader health team. If we want to build capacity in the workforce, consideration needs to be given to how professional development is funded. If allied health disciplines are only funded to deliver specific discrete services, ad hoc, there are limited opportunities or commitments to investment in upskilling the staff.

Key messages from these findings are that without sufficient funding, we risk losing a sufficient and expertly skilled workforce to deliver palliative and aged care services and measure the service outcomes to evaluate and inform best practice. The diversity in funding rules and streams across community and residential aged care services gives rise to inequity and inconsistent allied health input; across all settings where full scope of practice cannot be enacted within funding confines, experienced clinicians will seek career opportunities outside of the sector. All of this will compound on the care experience of the person at the centre of care, and it may only be the individuals who are located close to a range of services, with some private funding options and carer advocacy or capacity to initiate their own services, who receive allied health input in aged and palliative care.

## 4. Discussion

In summary, the issue of supporting allied health best practice in palliative care is multifactorial. At the centre seems to be the issue of how well recognised allied health disciplines are for the value of their contribution, and constraints imposed by funding models to be able to enact a full scope of practice. The aged care landscape is changing, and more older adults will want to receive ageing and palliative services in their own home. The stakeholders in this study were less confident to speak to home-based aged carers, other than for older adults with capacity, advocacy, and funds, for whom they perceive access to allied health services may be easier, presuming there is a sufficient workforce in aged care to service the need. However, a theme emerging in this study was that regional and remote areas are likely to be disproportionately impacted by a limited workforce in all settings. Such issues will become more urgent as changes to home-based aged care commence and include a specific end-of-life pathway as part of the reform funding model [16].

There was also a sense amongst interviewees that allied health professionals will need to be their own advocates in demonstrating the value of their input to continue conversations around adequate inclusions in funding models and involvement in multidisciplinary palliative care teams. It is particularly important that ‘gatekeeper’ referrers and clients have a good understanding of the services and benefits that allied health can provide and that AHPs are confident to articulate this. Currently, there is a paucity of the literature that discusses best practice for palliative allied health care in aged care [17]. Allied health disciplines will need to consider how best to develop and train health professionals to confidently collect and report on health outcomes, including quality of life measures and more nuanced person-centred outcomes for palliative care.

Person-centred care is building trust and rapport, and in palliative care, this is coupled with a high level of clinical reasoning and communication skills. The time usually afforded to allied health consultations often gives occasion for incidental conversations and a safe space for individuals to share fears or ask questions. Stakeholders are concerned that without investment in the time needed to deliver, review, and reflect on practice, allied health services to aged care will become transactional and infrequent and the workforce will lack capability to provide personalised care.

Strengthening education and training pathways for allied health professionals in palliative care is critical to ensuring high-quality, person-centred care. This includes embedding palliative care principles into pre-vocational curricula and expanding continuing professional development opportunities that address the complexity of end-of-life care. As an example, simulation-based training offers a practical and evidence-informed approach to building confidence and competence in managing sensitive scenarios, such as advanced communication, ethical decision-making, and responding to euthanasia requests. Research indicates that simulation enhances clinical reasoning and communication skills, which are essential in palliative contexts [18]. Alongside education, structured advocacy resources are needed to support allied health professionals in articulating their unique contributions to referrers, clients, and funding bodies. Furthermore, developing outcome frameworks that capture quality-of-life and maintenance-focused measures, rather than relying solely on improvement metrics, will strengthen the evidence base for allied health interventions. Finally, workforce strategies must prioritise regional and remote areas through telehealth integration and targeted incentives to address inequities in access and ensure that older Australians receive timely and comprehensive palliative care, regardless of location. Limitations to this study are acknowledged in the purposeful recruitment and, thus, present some risk of bias, and overall, the study is a limited snapshot of how the sector is reacting to aged care reform. However, the findings do also resonate with Palliative Care Australia National Standards for all health professionals (2022) [19] and a report exploring the value of allied health in residential aged care, commissioned by peak body AHPA (2021) [20]. The report recognises that with timely and effective allied health input, there are fewer emergency acute care admissions and an improved quality of life for older Australians. In addition, sufficient allied health workforce and involvement in care creates a more collaborative approach with other health providers or aged care workers, and support for family and carers supporting the older person. Although throughout this paper, allied health have been referred to collectively, it is important to note that each discipline can offer unique and specific skills and knowledge which speak to multiple standards of care within the strengthened aged care standards, including standard 5.7 palliative care [8].

## 5. Conclusions

Separating out the themes arising from the interviews has provided insight into where the ELDAC allied health toolkit may be well positioned to help address some of the practice and knowledge issues impacting person-centred care within funding constraints. However, the intent is that this research will also more broadly draw attention to the further work needed on how allied health can be supported to engage with the aged care sector to optimise care provision within the framework for funding and activity. How aged care services engage with allied health providers also needs to be considered to ensure that there is an opportunity to have education and training pathways for staff, to support visiting health professionals, and to integrate education and training in pre-vocational and continuing professional development requirements. These considerations are equally as important for residential aged care as home support. The risks of ignoring these issues will be an insufficient workforce to meet the demand and poorer outcomes for older adults in quality of life and death.

## Figures and Tables

**Table 1:** Overview of participant information.

Participant Code	Position	Location	Business Structure
P1	General Manager of Wellbeing and Allied Health Programs	SA	13 residential aged care sites, independent living, day therapy
P2	National Manager of Integrated Care Programs	SA, NSW, VIC, QLD, WA	29 residential aged care sites, independent living, 2 acute care facilities
P3	Aged Care Research and Industry Innovation Australia (ARIIA) Research Fellow/Physiotherapist	Australia-wide	Research centre addressing critical sector needs, supporting government initiatives and promoting collaboration and innovation in Australian aged care.
P4	Australian Allied Health in Palliative Care (AAHPC)—Committee Leadership/Occupational Therapist	Australia-wide	Professional association—to promote and support allied health professionals with an interest in palliative care
P5	Occupational Therapy Australia—Professional Practice and Aged Care Advisor/Occupational Therapist	Australia-wide	Member association—advocacy for Occupational Therapists (including in aged care/palliative care)
P6	Director—Allied Health Private Practice Provider: Podiatry	QLD/NSW	Home-based care—podiatry (including older adults)/previously also residential aged care
P7	Professor in Healthy Ageing Support and Care—Adelaide Primary Health Network/Occupational Therapist	SA	Supporting alignment and collaboration of research with primary health network commissioned ageing services
P8	ELDAC, Linkages Project Coordinator	Australia-wide	Building partnerships with aged care stakeholders for best practice

Key: SA = South Australia, NSW = New South Wales, VIC = Victoria, QLD = Queensland, WA = Western Australia, ELDAC = End of Life Directions for Aged Care.

**Table 2:** Key themes, sub-themes, and exemplar quotes from inductive theming of stakeholder transcripts.

Theme	Sub Theme	Exemplar Quote
Funding	“But look what I do think is that any, any reform that promotes the resourcing in aged care by allied health professionals also then allows those services to be delivered at end-of-life. so we don’t necessarily need to have structures or incentives in place specifically around end-of-life, but if we’ve just got more OT (occupational therapists) and physios and dietitians and social workers in aged care, then there will naturally provide a service into end-of-life” P1	
	Person-centred palliative care	“the common issues are really about … service provision and the focus of allied health and what they’ve got the time and the capacity for… the priorities are new residents, pain relief, ensuring that you do mobility assessment so people can be transferred safely with carers, and so the focus, umm, of those additional supportive services like rehabilitation, … like goal setting…you know that quality of care and finding out what activities for individuals are meaningful to support that as well as that whole extensive outside bit about social interaction which is important in all allied health but the time and the scope to be able to deliver that is certainly not there.” P3 “…one leg forward is like living the next leg forward is like dying… so I think acknowledging that those two things that, that’s a duality…of living and dying at the same time, and one doesn’t minimize the other…it can be really confronting because we’re reminding <them> their bodies are kind of falling apart and your job then is to support them in that distress as they process that” P4
	Advocates	“the other barrier that I think exists is a as a lack of awareness of the breadth of interventions that can be offered by allied health, and that that’s the allied health professionals themselves, but also, you know, nurses will still be the predominant referrer… So we’ve both sides don’t understand the value that can be provided or the different interventions then that’s also gonna limit good quality.” P1 “The individuals who have got families that care and are perhaps more involved in in them in aged care facilities and the community as well, who have got money and the means to get in a private therapist, certainly do better and get more of an input.” P3
	Location of Care Matters	“…in reality, if you live in residential aged care, your input and your likelihood of receiving rehabilitation is much reduced… So if you fall at home and you’re living at home and you go into hospital and have your… hip fracture fixed, you will receive rehabilitation and home care to get you back home… If you fracture your hip and you’re living in residential aged care, you’ll go and get that hip fixed in hospital and then potentially be discharged back to your residential care facility with no additional support… if you’re living in residential aged care, then why should the hospital fund that rehabilitation?… it’s sort of seen as double dipping if you’re trying to get rehabilitation when you’re already living in a high-level care service.” P3

## Data Availability

The original contributions presented in this study are included in the article. Further inquiries can be directed to the corresponding author.

## Abbreviations

The following abbreviations are used in this manuscript:

AHPA	Allied Health Professions Australia
AHPs	Allied Health Professionals
ELDAC	End of Life Directions for Aged Care
EOL	End-of-Life
DT	Dietitian
OT	Occupational Therapist
PT	Physiotherapist
SP	Speech Pathologist
